# The impact of young age (< 40 years) on the outcome of a cohort of patients with primary non-metastatic breast cancer: analysis of 10-year survival of a prospective study

**DOI:** 10.1186/s12885-021-09100-z

**Published:** 2022-01-03

**Authors:** Youssef Bouferraa, Yolla Haibe, Andrea Chedid, Elio Jabra, Maya Charafeddine, Sally Temraz, Deborah Mukherji, Nagi El Saghir, Ali Shamseddine

**Affiliations:** Department of Internal Medicine, Division of Hematology-Oncology, American University of Beirut Medical Center, Riad El Solh, Beirut, 1107 2020 Lebanon

**Keywords:** Breast cancer, Disease-free survival, Overall survival, Oncology, Age

## Abstract

**Background:**

The role of young age (< 40 years) at diagnosis as an independent risk factor for adverse outcomes in female patients with breast cancer has been highlighted in several studies. In this prospective study, we assessed the difference in 10-year survival between two groups of patients diagnosed with non-metastatic breast cancer based on an age cutoff of 40 years. We also assessed the impact of factors including tumor characteristics, molecular markers and immunohistochemical markers on survival outcomes, highlighting the interaction of those variables with age.

**Methods:**

A total of 119 female patients with newly diagnosed non-metastatic breast cancer were recruited at the American University of Beirut Medical Center (AUBMC) between July 2011 and May 2014. Patients were recruited and divided into 2 age groups (< 40 and ≥ 40 years). In addition to clinical characteristics, we assessed immunohistochemistry including estrogen, progesterone and HER2 receptors, p53, cyclin B1, vascular endothelial growth factor receptor (VEGFR), and ki-67. Germline BRCA mutations were also performed on peripheral blood samples. Patient and tumor characteristics were compared between the age groups. 10-year overall survival (OS) and disease-free survival (DFS) were estimated accordingly. Cox regression analysis was performed in order to assess the effect of the different variables on clinical outcomes.

**Results:**

After a median Follow-up of 96 (13–122) months, the estimated 10-year OS was 98.6% for patients ≥40 as compared to 77.6% in patients < 40 (*p* = 0.001). A similar trend was found for 10-year DFS reaching 90% for patients ≥40 and 70.4% for those < 40 (*p* = 0.004). On multivariate analysis for DFS and OS, only younger age (< 40 years), higher stage and triple negative phenotype among other parameters assessed significantly affected the outcome in this cohort.

**Conclusion:**

This prospective study confirms the association between younger age and adverse survival outcomes in patients with non-metastatic breast cancer. Future studies of the whole genome sequences may reveal the genomic basis underlying the clinical differences we have observed.

## Background

Breast cancer is the most common type of cancer worldwide, with an incidence of around 2.3 million cases in 2020 [1]**.** It also accounts by itself for 15.5% of cancer-related mortalities in females alone [1]. A similar epidemiologic profile is reported in Lebanon where breast cancer accounts for around one third of all reported cancer cases in females [2, 3]. While most breast cancer cases occur in older women, the worldwide incidence of the disease in the younger population has been on the rise in the past decade [4]. This is especially the case in Lebanon where around 20% of the breast cancer cases occur in females younger than 40 years old [5]. In particular, breast cancer in Lebanese female patients presents at a median age of 50 years, more than 10 years earlier than their female counterparts in the western countries where the median age at presentation approaches 63 years [6]. This increase in the incidence of the disease in the younger population has been attributed to several factors, including the increase in the mean marital age, the decrease in fertility, the increase in the prevalence of obesity and smoking in addition to the effect of awareness campaigns and mammography screening in Lebanon leading to earlier and more efficient detection [3, 4, 7]. These findings support the need to establish earlier screening guidelines in order to decrease the burden and the severity of breast cancer in the Lebanese women. In addition, they emphasize the necessity to study the risks and prognostic factors in the Lebanese patients with breast cancer below the age of 40 years.

The effect of age at diagnosis on the survival of patients with breast cancer is controversial and has been investigated in multiple studies worldwide. Contradictory results have been reported in the literature [5, 8–10]. Several prospective and retrospective studies concluded that young age at diagnosis constitutes an independent risk factor of poor survival outcomes in women with breast cancer [9, 11–15]. Using the Japanese Breast Cancer Registry between 2004 and 2006, Kataoka et al. found that young patients with breast cancer in Japan had lower disease-free survival (DFS) and overall survival (OS) rates, independent of other known clinicopathologic prognostic factors [11, 15]. Similar results were also described in Sweden and by our team in Lebanon, where younger age remained an independent risk factor for breast cancer death in women, especially in the early stages of the disease [5, 13]. Conversely, other studies reported no significant survival disadvantage in younger patients, with some going further and reporting better survival outcomes in the younger patient population [8, 10, 16].

As such, and in order to better understand the role of age at diagnosis on the outcome of patients with breast cancer in our population, we conducted a prospective study at a tertiary care and referral center in Lebanon involving Lebanese women recently diagnosed with non-metastatic breast cancer. We assessed the difference in 10- years survival outcomes between female patients with localized breast cancer aged below or above 40 years at diagnosis. We also studied the impact of several prognostic factors including molecular markers, genetic markers, and histopathologic markers on the survival outcomes, and the interaction of those variables with age.

## Methods

### Study design and patient recruitment

We conducted a prospective study at the American University of Beirut Medical Center AUBMC, a large tertiary care and referral center in Lebanon. We included newly diagnosed women with pathologically proven breast carcinoma between July 2011 and May 2014. In addition, we did not include patients with inflammatory cancer, cancer in situ, bilateral cancer, or a history of previous cancer. Our patients were classified into 2 age groups with a cutoff of 40 years. Patients were recruited after written informed consent was provided at the private oncology clinics or on wards at AUBMC. We used questionnaires in order to collect data regarding demographics, risk factors and medical history. We complemented the medical information by using clinical and hospital charts. We obtained survival data as patients were regularly seen and followed up on treatment wards and in clinics. In addition, we used telephone calls to follow up on patients. The study was designed and performed in accordance with the declaration of Helsinki. This study protocol was approved by the Institution Review Board (IRB) of the American University of Beirut (Study ID: IM.AS.17).

### Immunohistochemistry

Hematoxylin- and eosin-stained tumor sections were examined and tumor grades were defined using the Bloom-Richardson-Elston grading system [17]. Additional parameters including the presence of lymphovascular invasion or carcinoma in situ were also assessed. In addition, we performed immunohistochemical stains for ER, PR, HER2 and Ki67 in addition to molecular studies including p53, cyclin B1, and vascular endothelial growth factor receptor (VEGFR). Those stains were interpreted according to a grading system that takes into consideration for both the number of positive cells and the intensity of staining [18]. The latter was classified into weak, moderate or strong [18]. The number of positive cells were classified in 3 categories: < 5%, 5–50 and > 50% positive cells [18].

The 2011 St Gallen’s consensus was used to classify patients according to molecular subtypes [19]:

- Luminal A: ER and/or PR positive, HER2 negative, and Ki-67 < 14% or low/intermediate grade
- Luminal B (combined as 1 subtype):
  - ◦ Luminal B (HER2 negative): ER and/or PR positive, HER2 negative, and Ki-67 > 14% or high grade
  - ◦ Luminal B (HER2 positive): ER and/or PR positive, any Ki-67, and HER2 amplified or overexpressed
- Erb-B2 overexpression: ER negative, PR negative, and HER2 positive
- Triple negative: ER negative, PR negative and HER2 negative

### Genetic analysis

We extracted DNA by using whole blood samples. Sanger technique was used to analyze for germline BRCA1 and BRCA2 mutations.

### Statistical analysis

Frequencies were used to describe the population, while for cross tabulations the Pearson chi-square was used to compare the 2 age groups (age <  40 and age ≥ 40) in terms of treatment, outcomes, tumor characteristics, pathological variables, and molecular variables. Fisher’s exact test was used when the number of cases was low (less than 5%) in any of the cross tabulation cells in order to account for the low number of patients < 40 years old. The student’s t-test was used to compare mean sizes of the tumors between the two groups. Disease free survival time (DFS) was calculated from the date of diagnosis to the date of relapse or to the date of last follow-up visit or telephone call in case no recurrence was documented (censored observation). Similarly, overall survival time (OS) was calculated from the date of diagnosis to the date of death or to the date of last follow-up visit or telephone call in case no death was documented (censored observation). Patients who were lost to follow up were included in the analysis until their last contact with the research team either over a phone call or a visit. DFS and OS curves were plotted using the Kaplan-Meier curves; log rank was used to check for significant difference between the 2 age groups. Multivariate analysis for DFS was done using Cox-regression analysis. The following variables were included in the model: Triple negative, luminal A, luminal B, surgical margins, BRCA status, age groups (age <  40 and age ≥ 40), grade and stage using the backward stepwise conditional regression analysis which keeps the significant variables only in the final model. We evaluated the hazard ratio and its 95% confidence interval for each variable. We used *p* < 0.05 to define statistical significance. All analyses were done using the SPSS version 25.0.

## Results

Between July 2011 and May 2014, a total of 119 patients were recruited into this study. The median age of all patients was 44 years (27–76) years. 73 (62%) patients were 40 years of age or older with a median age of 51 (41–76) years. 46 (39%) patients were below the age of 40 years with a median age of 36.5 (27–39) years (Table 1). 32% of the patients presented with stage I disease, 45% presented with stage II disease, and 23% of the patients had stage III disease.. When stratified by age, 60% of the patients ≥40 years presented with stage I or stage II disease as compared to 32% in the younger < 40 years population (Table 1). When divided into subtypes, luminal A subtype was the most common subtype in both age groups, constituting 39% in the cohort < 40 years as compared to 44% in the cohort ≥40 years (Table 1).

**Table 1:** Patient characteristics

% (n)	Age above 40 (***n*** = 73)	Age below 40 (***n*** = 46)	***p***-value
**Marital status**	78.1 (59)	82.6(40)	0.457
**Breast feeding**	75.8 (50)	76.7 (33)	1
**Physical activity**	37 (27)	28.3 (13)	0.426
**Vegetable and fruits**	80.8 (59)	82.2 (37)	1
**Calcium intake**	21.9 (16)	8.9 (4)	0.081
**VIT D intake**	24.7 (18)	13 (6)	0.161
**Recurrence**	9.6 (7)	28.3 (13)	0.012
**Smoker**	49.3 (36)	37 (17)	0.256
**Alcohol intake**	15.1 (11)	19.6 (9)	0.346
**Positive Surgical margins who ever have positive margins, re-excision was done to render margins negative**	10 (7)	19 (8)	0.251
**Grade**			0.824
**1**	19.2 (14)	15.6 (7)	
**2**	45.2 (33)	44.4 (20)	
**3**	35.6 (26)	40 (18)	
**cT size**			0.018
**T1**	47.9 (35)	37 (17)	
**T2**	45.2 (33)	41.3 (19)	
**T3**	2.7 (2)	19.6 (9)	
**T4**	4.1 (30	2.2 (1)	
**cNode status**			0.332
**N0**	57.5 (42)	44.4 (20)	
**N1**	28.8 (21)	40 (18)	
**N2**	13.7 (10)	15.6 (7)	
**Stage**			0.099
**1**	38.4 (28)	21.7 (10)	
**2**	43.8 (32)	47.8 (22)	
**3**	17.8 (13)	30.4 (14)	
**Erb2 positive**	6.9 (5)	8.7 (4)	0.735
**Ki67**			0.140
**< 10%**	19.4 (12)	26.3 (10)	
**10–20%**	33.9 (21)	15.8 (6)	
**> 20%**	46.8 (29)	57.9 (22)	
**Hormone receptor positive**	87.7 (57)	80 (28)	0.381
**Luminal A**	44.4 (32)	39.1 (18)	0.703
**Luminal B**	34.7 (25)	26.1 (12)	0.218
**Triple negative**	13.9 (10)	26.1 (12)	0.378
**ERB2**	6.9 (5)	8.7 (4)	0.735
**Mastectomy**			0.087
**Total**	39.7 (29)	56.8 (25)	
**Partial**	60.3 (44)	43.2 (19)	
**Family history breast cancer**	50.7 (37)	52.2 (24)	1
**BRCA**	7.2 (5)	7.9 (3)	1

**Fig. 1:**
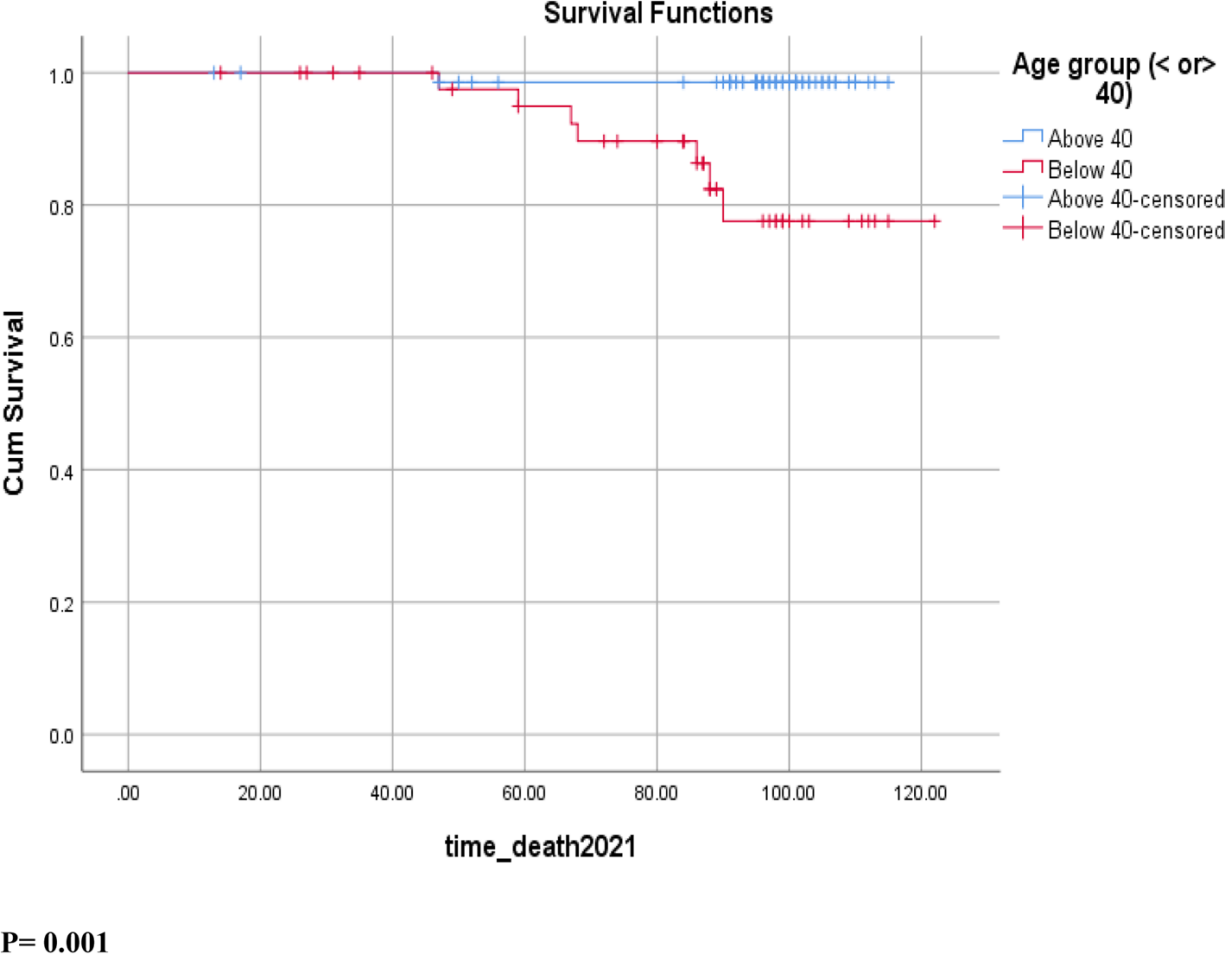
Overall Survival OS in breast cancer patients stratified by age groups

**Fig. 2:**
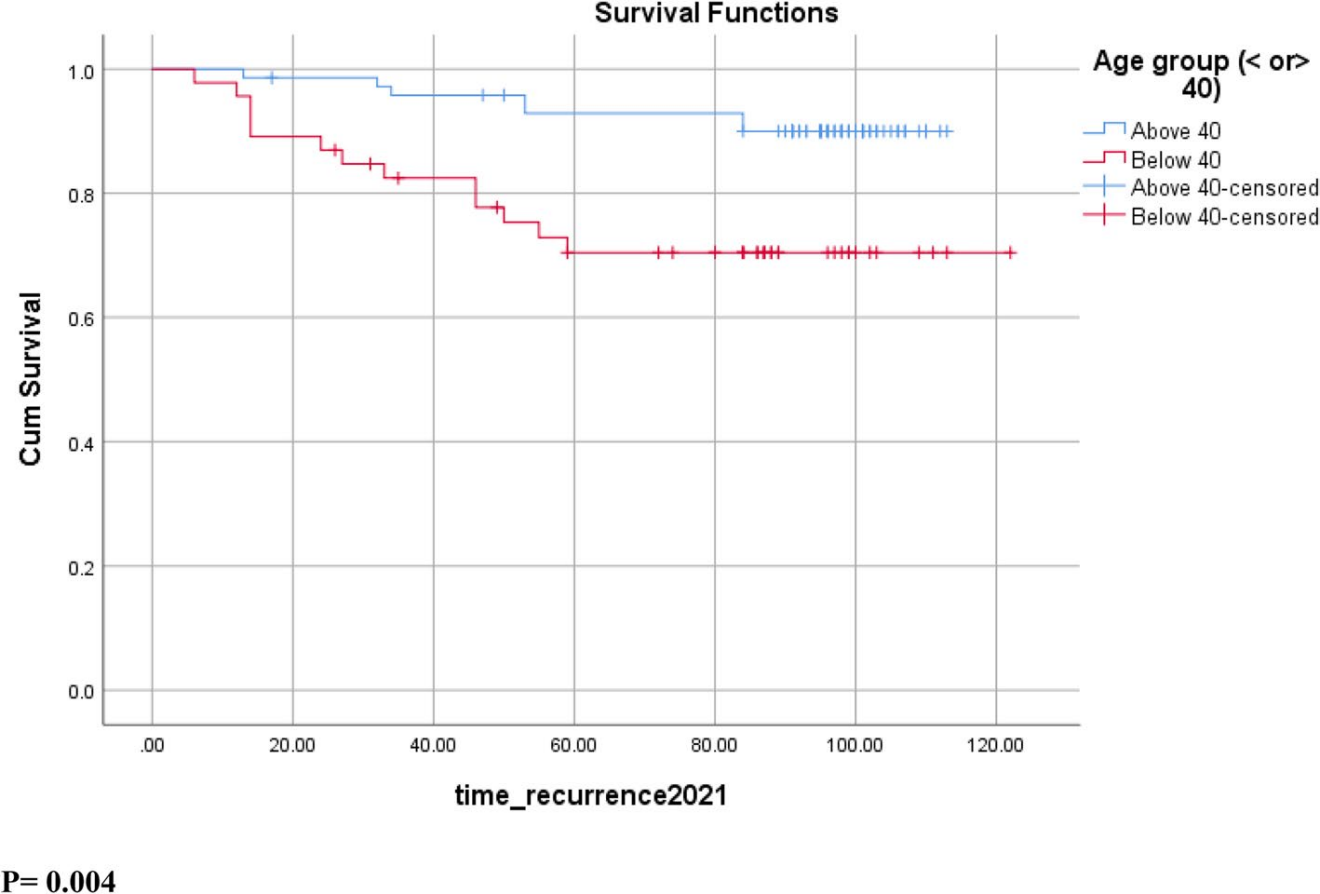
Disease-Free Survival DFS in breast cancer patients stratified by age groups

The median follow-up period was 96 months, ranging from 13 to 122 months. The median follow-up period was 87 months for patients < 40 years and 98 months for those above 40 years. The estimated 10-year OS was significantly higher in the population older than 40 years old, being 98.6% for patients ≥40 as compared to 77.6% in patients < 40 (*p* = 0.001) (Fig. 1). A similar trend was found with 10-year, being 90% for patients ≥40 and 70.4% for those < 40 (*p* = 0.004) (Fig. 2). A similar trend was found for 10-year OS, reaching 98.6% for patients ≥40 as compared to 77.6% in patients < 40 (*p* = 0.001) (Fig. 2). The mean time to recurrence (mean DFS) was 95 months for patients < 40 compared to 107 months for patients above 40 years of age (*p* = 0.004).

**Fig. 3:**
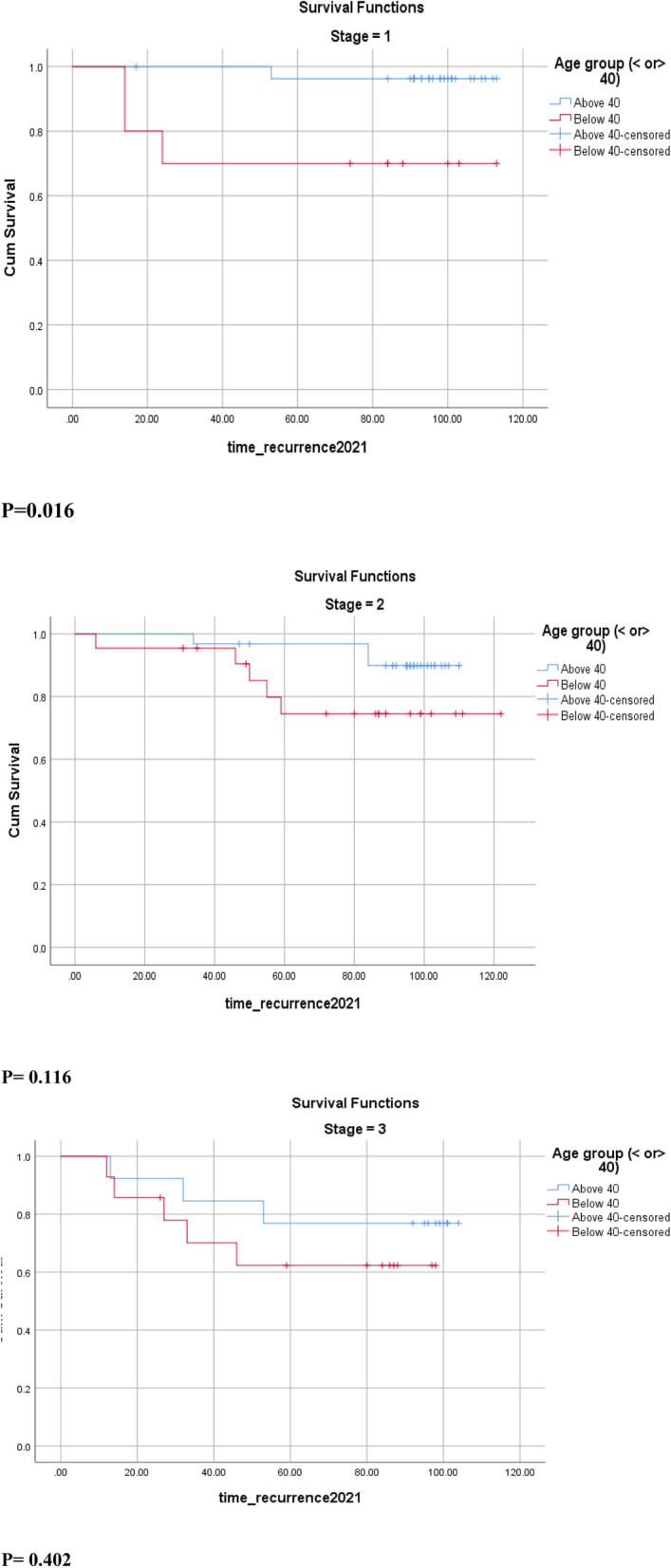
Disease-Free Survival DFS in stage I, II and III breast cancer patients stratified by age groups

Patients in the age group below 40 had a higher percentage of recurrence in all disease stages, stage I in particular (30% vs 3.6%) (Fig. 3) (Table 2). Stage III patients had the highest recurrence rate with 29.6% compared to 15.1% in stage II and 10.5% in stage I patients (*p* = 0.026). Patients older than 40 years of age were more likely to present with T1 (47.9% vs 39%, *p* = 0.018), which could be attributed to screening (Table 1). Additionally, for the same T1, the cohort < 40 years had a higher chance of recurrence (17.6% vs 2.9%), but the result was non-significant (*p* = 0.097).

**Table 2:** Recurrence rates for the different stages stratified by age groups

% (n)	Age < 40	Age ≥ 40	All patients
**Stage 1**	30.0 (3)	3.6 (1)	10.5 (4)
**Stage 2**	22.8 (5)	9.7 (3)	15.1 (8)
**Stage 3**	35.7 (5)	23.1 (3)	29.6 (8)
**All stages**	28.3% (13)	9.7% (7)	16.9 (20)

The prevalence of BRCA mutation was found to be similar between the 2 age groups, being 7.9% in patients < 40 years and 7.2% in patients ≥40 years (*p* = 1) (Table 1). In addition, 25% of the patients with triple negative disease had a BRCA1 or a BRCA2 mutation as compared to only 3.5% in the remaining types of tumors (*p* = 0.006) (Table 3). Other pathologic features including grade of differentiation, lymph node status, hormone receptor expression and surgical margins’ positivity, were similar between the 2 age groups and showed no effect on the disease recurrence in our patients. No difference was observed between the various immunohistochemical characteristics studied (cyclin B1, p53, VEGFR, Ki67) between age groups or on possibility of recurrence (Table 1). Additionally, several patients’ attributes were also studied including alcohol intake, smoking, physical activity, vitamin C and D intake, and they showed no impact on risk of recurrence irrespective of the age (Table 1).

**Table 3:** Prevalence of BRCA gene mutation in triple negative disease

	BRCA Mutation		
Triple Negative Disease	No	Yes	Total
**No**	96.5 (83)	3.5 (3)	100 (86)
**Yes**	75 (15)	25 (5)	100 (20)

Most patients with positive family history presented with stage I disease but the difference between the 2 age groups was not significant (Table 4). In addition, less recurrence was seen in all patients with positive family history with a rate of 11.5% vs 22.4%, (*p* = 0.031) (Table 5). When stratifying by age, and in patients with a family history of breast cancer, 25% of the age group below 40 years had recurrence of the disease as compared to 0.03% in their older counterparts (*p* = 0.020) (Table 5).

**Table 4:** Disease stages stratified by family history of breast cancer

% (n)	Stage 1	Stage 2	Stage 3	Total
**Presence of family history**	41 (25)	39.3 (24)	19.7 (12)	100 (61)
**Absence of a family history**	22.4 (13)	51.7 (30)	25.9 (15)	100 (58)
**Total**	31.9 (38)	45.4 (54)	22.7 (27)	100 (119)

**Table 5:** Disease recurrence stratified by age and family history of breast cancer

		Recurrence rate % (n) ***p*** = 0.031		
Family History	Age Group	No	yes	Total % (n)
**Presence of a family history** ***P*** **= 0.005**	**< 40**	75 (18)	25 (6)	100 (24)
	**> 40**	97.3 (36)	0.03 (1)	100 (37)
	**Total**	88.5 (54)	11.5 (7)	100 (61)
**Absence of a family history** ***P*** **= 0.124**	**< 40**	68.2 (15)	31.8 (7)	100 (22)
	**≥**	83.3 (30)	16.7 (6)	100 (36)
	**Total**	77.6 (45)	22.4 (13)	100 (58)

When looking at surgery types, partial mastectomy was the most common surgery in patients above 40 as compared to radical mastectomy in patients below 40 (*p* = 0.087), but the difference was only statistically significant in patients with T1 disease (Table 6) (*p* = 0.021). No difference was noted between the 2 age groups in terms of neoadjuvant and adjuvant therapies, including chemotherapy, Herceptin, hormonal and radiation therapy.

**Table 6:** Surgery type in patients with T1 disease stratified by age group

Age	Radical Mastectomy % (n)	Lumpectomy % (n)	Total % (n)
**< 40**	56.3 (9)	43.8 (7)	100 (16)
**≥**	20 (7)	80 (28)	100 (35)
**Total**	31.4 (16)	68.6 (35)	100 (51)

Using Cox regression model, the multivariate analysis for DFS showed that younger age (< 40 years), higher stage and triple negative disease remained significant in the model controlling for all other variables including grade and molecular subtypes which were not significant in the model. Patients less than 40 years had 4.4 times the risk of their older counterparts 95%CI (1.33–14.69), *P* = 0.016). Stage was significant in the model with a risk of 2.16 95%CI (0.603–7.74) for stage III patients compared to stage I, although the p- value was not significant for stages II and III compared to stage I. Patients with triple negative disease were at higher risk of developing disease progression with a hazard ratio of 3.13 (1.01–9.73), *p*-value =0.048.

## Discussion

Breast cancer in young women is considered to have a worse prognosis because of multiple factors, including its biological characteristics, aggressive clinical behavior, and late stages at presentation [20]. This is the first prospective study in Lebanon with a long follow-up period reporting significantly lower 10-year survival outcomes in patients with non-metastatic breast cancer < 40 years as compared to patients ≥40 years. In our study, patients younger than 40 years old were found to have significantly lower 10-year OS (77.6% vs 98.6% in < 40 and ≥ 40 respectively, *p* = 0.001) and 10-year DFS (70.4% vs 90% in < 40 and ≥ 40 respectively; *p* = 0.004) (Figs. 1 & 2). These results were statistically significant and younger age (< 40) remained an independent prognostic factor for worse survival in patients with breast cancer after adjusting for other clinicopathologic prognostic factors. Other studies previously found similar results in the Lebanese population. In a retrospective study between 1990 and 2001, and with a median follow-up period of 2.9 years, El Saghir et al. reported a negative impact of young age on the survival outcomes in the Lebanese patients with breast cancer [9]. Similar results were also published in 2017 in a prospective study from the same cohort of patients after a median follow up of 58 months with a significantly lower 5-year DFS in patients < 40 year (*p* = 0.03) [5].

Several explanations have been raised in the literature in an attempt to investigate the effect of age on the survival of patients with breast cancer. Many studies have concluded that young patients with breast cancer usually present with advanced stages [21–24]. In a sub-analysis of the AMAZON III study in Brazil, it was shown that younger patients with breast cancer were more likely to present with stage III disease as compared to stage I in their older counterparts [21]. These findings were also detected in different studies conducted in Turkey and Argentina where stage III breast cancer was more frequently seen in young patients [22, 24]. This could be partially explained by the fact that younger patients are more likely to go undetected until they develop symptoms, which usually occurs at more advanced stages of the disease [21]. In our study, while stage III was more common in patients < 40 years (30.4%), and while stage I was more frequently encountered in ≥40 years (38.4%), these results were not statistically significant, and no conclusion could be raised regarding the higher prevalence of advanced stages at presentation in our young population (< 40 years) (Table 1). In all cases, stage remained an independent factor for disease-free survival, and stage III patients had the highest rates of recurrence. In addition, patients below 40 had higher rates of recurrence in all stages of the disease as compared to those older than 40 years (Table 2). Moreover, several published articles have found that younger patients usually have larger tumors at diagnosis due to lower screening rates in this population [21, 22, 25]. In our cohort, when classified into T categories, T1 was the most common size in patients above 40 as compared to T2 in those below 40, and the difference was statistically significant (*p* = 0.018) (Table 1).

In addition to stage and tumor size, other prognostic histopathologic features were investigated in the literature order to explore the worse survival outcomes in the younger patient population [26–29]. Younger patients with breast cancer were reported in the literature to have higher grades of tumor differentiation, axillary lymph node involvement and HER-2 expression [14, 26–29]. In this study, no statistically significant difference was noted in terms of histologic type, grade, lymph node involvement, lymphovascular invasion, surgical margin positivity, hormone receptor positivity, Her2/Neu receptor expression, ki-67, p53, cyclin B1, and VEGFR expression between the 2 groups (Table 1).

Moreover, different breast cancer subtypes are known to have distinct rates of cancer recurrence and mortality [30]. Luminal type A subtype was found to have the lowest rate of recurrence as opposed to triple negative subtype that recurs the most [30–33]. In addition, the effect of age on the survival of patients with breast cancer was also found to be affected by the tumor subtype, with the poor prognostic factor of younger age (< 40 years) mostly detected in luminal cancer subtypes [15, 34]. In our present study, luminal A subtype was the most common in both age groups, with a higher prevalence in patients above the age of 40, but the difference was not significant. (Table 1).

The prevalence of BRCA mutations was found to be low and nearly similar between the 2 age groups (Table 1). These results are consistent with previously reported data by El Saghir et al. who noted that the prevalence of deleterious BRCA mutations in patients at high risk of having hereditary breast cancer was 5.6% in the Lebanese population, which is lower than what is seen in similar French patients. As such, BRCA mutations do not explain by themselves the high incidence and recurrence of breast cancer in the young Lebanese and Arab population [35]. However, patients with triple negative disease had a significantly higher incidence of BRCA mutation as compared to patients with the remaining types of tumors, even in the absence of a family history of disease (Table 3). The NCCN guidelines recommend testing for BRCA mutation in patients with triple negative disease up until the age of 60 [36].While we acknowledge our small sample size, these results support the recommendations of BRCA genetic testing in patients with triple negative tumors regardless of age and family history, as proposed by other studies [37].

**Fig. 4:**
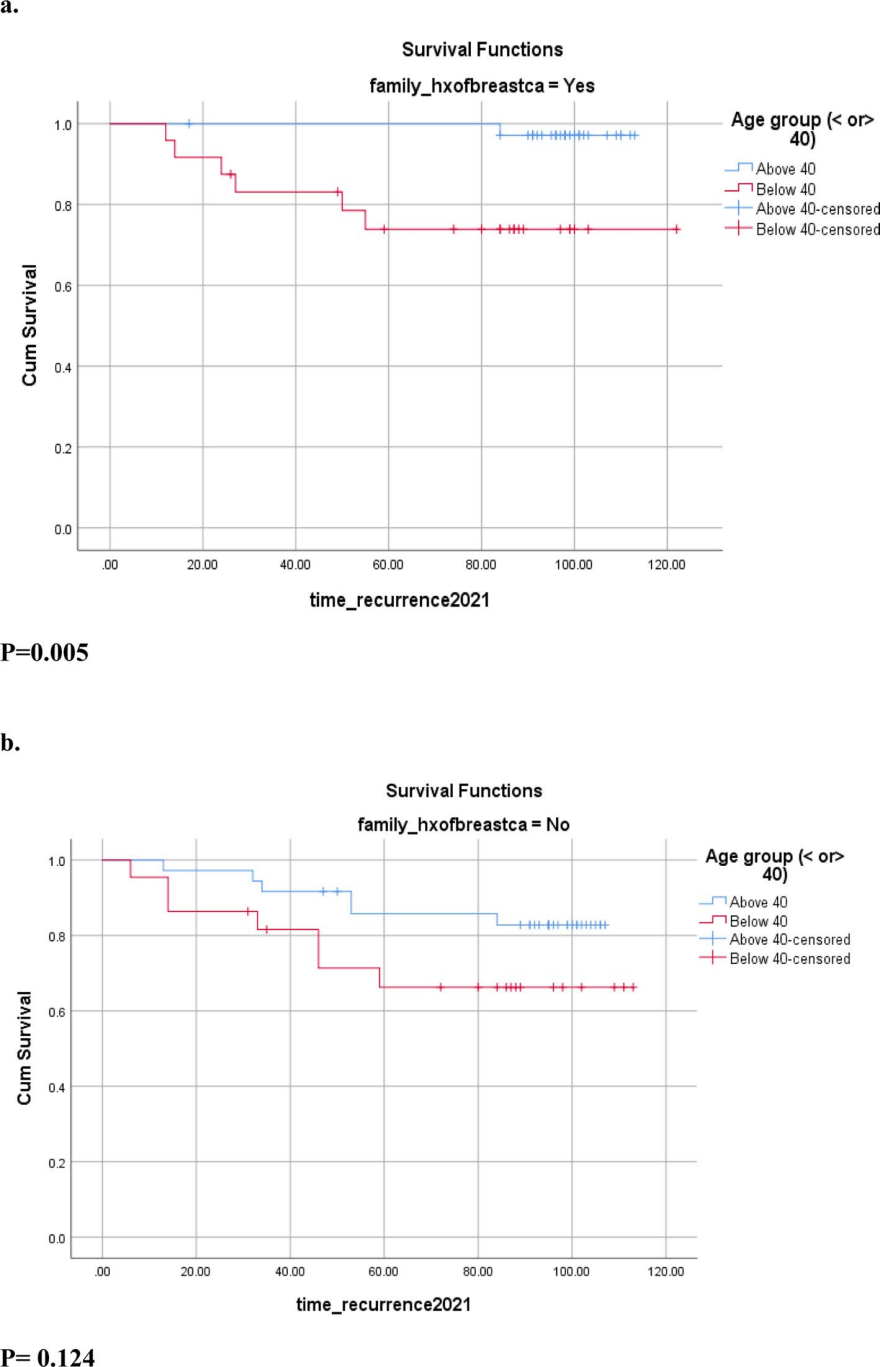
Disease-Free Survival DFS in patients with (**a**) and without (**b**) family history of breast cancer stratified by age groups

We also noted in our study a statistically significant lower rate of disease recurrence in patients with a family history of breast cancer, being 11.5% in this population as compared to 22.4% in those without a family history of the disease (*p* = 0.031) (Table 5). Many previous studies suggested that family history of breast cancer increases the risk of breast cancer recurrence [38–40]. On the other side, several other reports denied this relationship, with some associating the presence of a family history with a decreased rate of local recurrence [41–43]. Fourquet et al. suggest that this difference might be explained by an increased sensitivity of the population with a family history of breast cancer to radiotherapy [44]. Age also had an impact on the relationship between family history and recurrence. In this study, taking patients with a positive family history of breast cancer, we noticed that the patients below the age of 40 had a significantly higher rate of disease recurrence as compared to the patients above the age of 40 (*p* = 0.05) (Table 5) (Fig. 4). This finding further stresses on the importance of young age at diagnosis as a negative prognostic factor in breast cancer patients.

In terms of treatment, no significant difference in rates of chemotherapy, radiotherapy or surgery frequency was noted between the 2 age groups. However, when stratifying in terms of surgery type and disease stage, we noticed that patients less than 40 years of age with T1 disease were more likely to undergo a radical mastectomy as compared to a partial mastectomy in their older counterparts (≥40 years) with similar disease, and the difference was statistically significant (Table 6). Similar results were reported in the United States where a National Cancer Data Base study reported an increased trend of mastectomy over BCT in young patients with breast cancer, despite the lack of evidence suggesting the superiority of the former [45, 46]. This might be in part explained by the fact that young age has been considered by care providers as a negative survival prognostic factor pushing them towards adopting a more aggressive treatment approach. In addition, young patients are more likely to be involved in their healthcare-related decisions, with many opting towards radical aggressive treatments for different reasons including perceived benefit of improved survival, “peace of mind”, fear of the need of a repeated surgery or even inability to comply with daily post-BCT radiotherapy because of multiple familial and occupational commitments [45, 47–49]. No survival benefit and lower quality of life were reported in several studies comparing mastectomy and BCT in young patients [45, 50, 51]. While they acknowledge the increased risk of recurrence in the younger population in their review, Beadle et al. argue that the data is not strong enough to recommend that young age alone should constitute an indication for mastectomy alone [52]. They conclude that decision should be individualized for each patient taking into consideration the histologic and molecular subtypes of each tumor [52].

Our study has some limitations that need to be acknowledged. Our sample size is relatively small and studies with larger patient populations are needed to increase the power of the study. In addition, treatment modalities were not standardized between the patients of this study. While no statistically significant difference was noted between the treatment modalities used in the 2 age groups, the absence of detailed information about the neoadjuvant and adjuvant therapy types and duration constitutes a limitation to our study. Moreover, our study did not assess the number of patients who had their cancer diagnosed by clinical symptoms versus radiologic screening in each arm, as patient who present with symptomatic disease usually have worse prognosis. Besides, while no statistically significant difference was noted in several patients’ attributes (including alcohol intake, vegetable and fruit intake, smoking, physical activity, calcium, vitamin C and D intake) between the 2 age groups, our data lack the detailed information about the socio-economic status of the patients in both age groups. Finally, no genome sequencing of tumors was done in order to elicit any significant genomic differences between the 2 groups.

## Conclusion

In conclusion, this study sheds the light about the role of younger age (< 40 years) as a negative prognostic factor in the survival of patient with breast cancer. While many explanations of this observation have been raised, no definitive answer regarding the exact mechanism through which younger age (< 40 years) negatively affects survival in patients with breast cancer has been established. As most parameters including tumor characteristics, patients’ attributes and family history were similar between the cohorts in this study, a prospective study with whole genome sequences may shed the light and explain the difference of survival outcomes between the two groups.

## Data Availability

The datasets generated and analyzed during the current study are not publicly available for ethical purposes but are available from the corresponding author on reasonable request.

## Abbreviations

AUBMC: American University of Beirut Medical Center
DFS: Disease-free survival
IRB: Institution Review Board
OS: Overall survival
VEGF: Vascular endothelial growth factor
